# Endovascular Mechanical Thrombectomy for Right Hemispheric Stroke Syndrome Due to Acute Left A1-A2 Junction Thromboembolic Occlusion

**DOI:** 10.31486/toj.23.0042

**Published:** 2023

**Authors:** Tyler Scullen, James Milburn, Mansour Mathkour, Peter S. Amenta

**Affiliations:** ^1^ Department of Neurological Surgery, Tulane University, New Orleans, LA; ^2^ Department of Neurological Surgery, Ochsner Clinic Foundation, New Orleans, LA; ^3^ Department of Radiology, Ochsner Clinic Foundation, New Orleans, LA; ^4^ The University of Queensland Medical School, Ochsner Clinical School, New Orleans, LA; ^5^ Department of Neurological Surgery, University of Massachusetts Medical School, Worchester, MA

**Keywords:** *Endovascular procedures*, *intracranial embolism*, *mechanical thrombolysis*

## Abstract

**Background:** Endovascular mechanical thrombectomy (EVT) for large vessel occlusions has had a dramatic impact on the management of acute ischemic stroke. Extended use of EVT beyond American Heart Association guidelines has been successful in carefully selected cases.

**Case Report:** A 71-year-old male presented to our comprehensive stroke center upon awakening with mild left hemiparesis. He was found to have a chronic occlusion of the right supraclinoid segment of the internal carotid artery. Angiography demonstrated large vessel occlusion of the contralateral A1-A2 junction that was successfully recanalized. Imaging at 24 hours displayed no evidence of infarct, the patient rapidly improved during hospitalization, and he was discharged on postoperative day 7 with a National Institutes of Health Stroke Scale score of zero.

**Conclusion:** We describe successful EVT of a patient presenting with false-localizing symptoms consistent with a right hemispheric acute ischemic stroke secondary to left A1-A2 junction large vessel occlusion. This case demonstrates the importance of a high index of suspicion when evaluating atypical stroke presentations and the effectiveness of EVT in the treatment of distal small-caliber vessels.

## INTRODUCTION

Endovascular mechanical thrombectomy (EVT) for large vessel occlusion has dramatically impacted the management of acute ischemic stroke.^1-3^ Concurrently, the establishment of comprehensive stroke centers has allowed for rapid triage, transportation, and treatment.^1-3^ Accordingly, indications for EVT are a subject of intense academic focus and optimization, as the American Heart Association guidelines for the treatment encompass roughly 30% of large vessel occlusion patients^4^ presenting to a given comprehensive stroke center.^5,6^ Multiple reports have been published evaluating the extended use of emergent recana-lization, including applications beyond the primary arterial tree to treat medium vessel occlusions, thereby broadening the inclusion criteria and extending the timelines of patients who may benefit from treatment in appropriate clinical scenarios.^7-30^

We describe a case of false-localizing right hemispheric stroke syndrome due to an atypical acute left anterior cerebral artery A1-A2 junction thromboembolic large vessel occlusion in the setting of a chronic right internal carotid artery occlusion. The patient was successfully treated with emergent EVT and made a complete recovery.

## CASE REPORT

**Figure 1.**
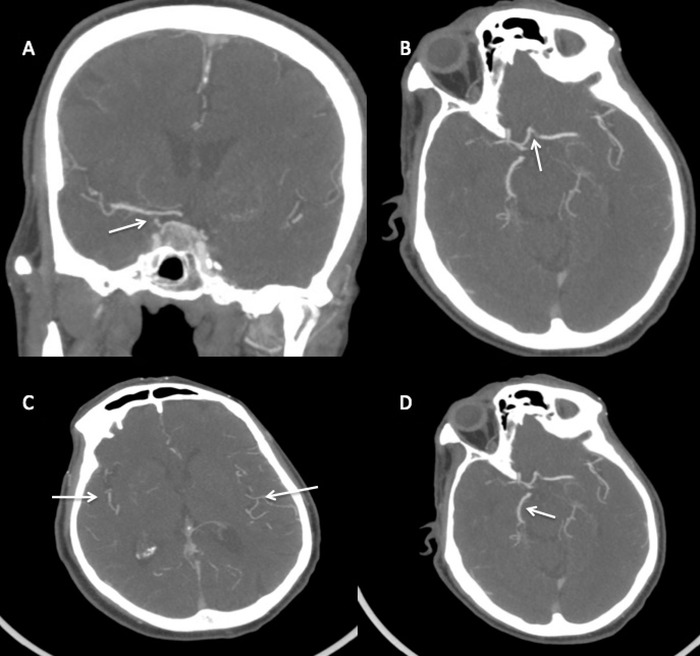
(A) Coronal computed tomography (CT) angiography of the head demonstrates short segment occlusion of the right supraclinoid segment of the internal carotid artery (arrow). (B) Axial view shows collateral flow from contralateral anterior circulation across the anterior communicating artery (arrow). (C) The bilateral middle cerebral arteries fill completely on axial CT angiography without branch occlusions (arrows), as does (D) the right posterior cerebral artery through the anterior communicating artery (arrow).

**Figure 2.**
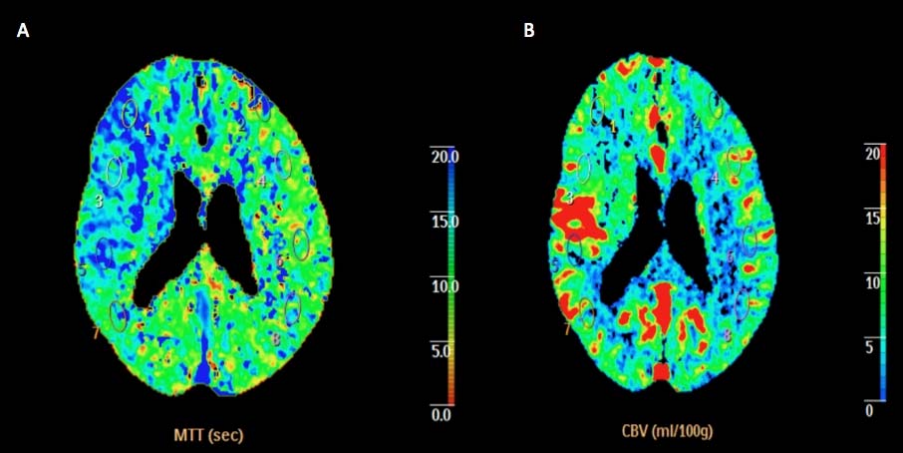
Computed tomography perfusion scan of the brain shows (A) increased mean transit times (MTT) in right middle cerebral artery territory and (B) normal symmetric cerebral blood volumes (CBV), indicative of a large penumbra.

A 71-year-old male with a history of cardioverter-defibrillator placement and atrial fibrillation on apixaban presented to our comprehensive stroke center upon awakening with left-sided weakness. Examination on arrival demonstrated mild left hemiparesis and a National Institutes of Health Stroke Scale (NIHSS) score of 4. Computed tomography (CT) angiography demonstrated total occlusion of the right supraclinoid segment of the internal carotid artery (Figure 1). Distal to the occlusion, complete filling of the anterior cerebral artery and middle cerebral artery territories suggested significant collateralization, speculatively via the anterior communicating artery complex (Figure 1). While the right A1, anterior communicating artery, and the left A1-A2 junction appeared to opacify with contrast, the tortuous left A1-A2 junction was difficult to definitively evaluate. CT perfusion demonstrated a large right hemispheric penumbra with preserved blood volume (Figure 2).

The patient was not a candidate for intravenous tissue plasminogen activator secondary to therapeutic anticoagulation and recent upper gastrointestinal bleeding. Following imaging, the patient acutely deteriorated, developing dense left hemiplegia, severe dysarthria, and a fixed right gaze (NIHSS score of 25), consistent with right hemispheric infarct. Neurosurgery was consulted for emergent EVT.

**Figure 3.**
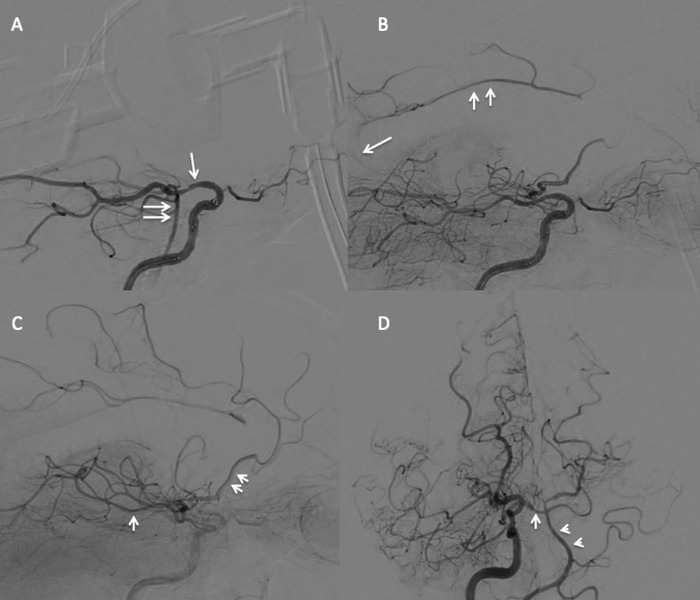
Lateral digital subtraction angiography (DSA) of the right internal carotid artery shows (A) chronic occlusion of the communicating segment of the right internal carotid artery (arrow) and retrograde flash filling of the basilar artery (double arrows) via the posterior communicating artery. (B) Mid-arterial phase shows leptomeningeal collateral flow from the splenial branch of the posterior cerebral artery (arrow) to the pericallosal branch of the anterior cerebral artery (double arrows) that fills retrograde, as well as leptomeningeal collateralization (C) between the middle cerebral artery (arrow) and proximal anterior cerebral artery (double arrows). (D) Anteroposterior DSA right internal carotid artery injection shows dominance of the internal carotid artery in perfusing the vertebrobasilar system (double arrowheads) via retrograde flow of the right posterior cerebral artery P1 segment (arrow).

Transfemoral access was gained, and the right internal carotid artery was selected with a 5 French (F) vertebral catheter (Cook Medical) through a 6F-80 shuttle sheath (Cook Medical). Digital subtraction angiography demonstrated a chronic intracranial internal carotid artery occlusion distal to the origin of the right anterior choroidal artery. The late arterial phase showed right ipsilateral leptomeningeal collateralization from the posterior cerebral artery to the anterior cerebral artery and middle cerebral artery posterior division (Figure 3).

**Figure 4.**
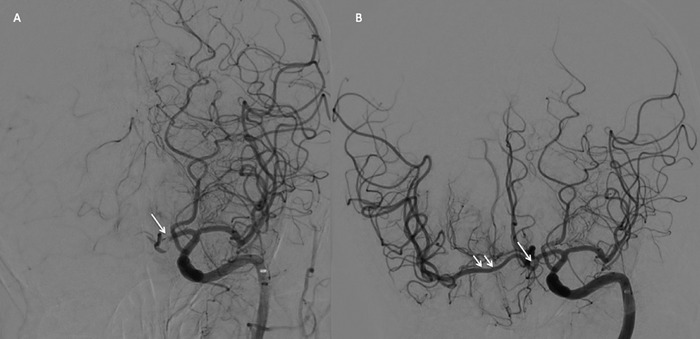
Anteroposterior digital subtraction angiography of the left internal carotid artery demonstrates (A) acute occlusion of the left A1-A2 junction (arrow). (B) Postthrombectomy injection demonstrates recanalization of left A1-A2 junction, bilateral anterior cerebral arteries, and bilateral middle cerebral arteries (arrows).

Given the patient's acute neurologic deterioration, the left internal carotid artery was selected to confirm etiology, demonstrating large vessel occlusion of the left A1-A2 junction by acute thrombus that was not clearly seen on CT angiography (Figure 4). Superselective catheterization of the left A2 past the thrombus was performed with a Headway 0.021-in microcatheter (MicroVention, Inc) and Synchro^2^ standard 0.014-in microwire (Stryker Corporation), and EVT was undertaken via 4 × 40 Solitaire stent (Medtronic) deployment across the A1-A2 junction. Following 5 minutes of radial incorporation, the device was withdrawn under aspiration through a SOFIA 6F-125 catheter (MicroVention, Inc). Postthrombectomy injection confirmed total recanalization, thrombolysis in cerebral infarction score of 3, and patency of the bilateral middle cerebral artery and bilateral anterior cerebral artery territories (Figure 4). Noncontrast head CT at 24 hours displayed no evidence of infarct, and the patient rapidly improved during hospitalization. The patient was discharged to an inpatient rehabilitation facility on postoperative day 7 with an NIHSS score of zero.

## DISCUSSION

Our patient had an occlusion of the left A1-A2 junction, resulting in a presentation consistent with a right M1 large vessel occlusion. Initial imaging supported his neurologic clinical findings, as the right supraclinoid segment of the internal carotid artery was shown to have an intracranial occlusion of unknown chronicity. The left A1-A2 junction demonstrated significant tortuosity, and the occlusion was not clearly identified on CT angiography. Access across the left A1-A2 junction and successful EVT using a stentriever resulted in complete recanalization of bilateral anterior cerebral arteries and bilateral middle cerebral arteries, allowing complete neurologic recovery.

This case highlights multiple critical aspects in the management of large vessel occlusion, the first of which is the importance of a high index of suspicion in patients with acute deterioration. Superficial consideration of the clinical picture, the patient's known cardiac history, and a clear chronic right internal carotid artery occlusion could have been incorrectly attributed to a hypoperfusion event and led to abortion of EVT prematurely.^18-20,23,24,27^ Because of the profound change in the patient's neurologic examination, a more aggressive workup was required and ultimately led to the correct diagnosis and intervention.

Additionally, the outcome achieved in this case further demonstrates the utility of EVT in select patients who fall outside of American Heart Association guidelines, particularly those with extended or intervally progressing windows of symptom onset and distal occlusions.^5^ While EVT has been shown to be an effective and safe treatment in the management of acute large vessel occlusion for patients presenting within 6 hours of symptom onset,^21^ academic efforts have expanded the clinical utility in subselected patients with low Alberta Stroke Program Early CT Scores,^22^ presenting at delayed time intervals (as evidenced by continued analysis in the recent DAWN and DEFUSE-3 trials),^22,23^ and with distal occlusions and medium vessel occlusions (reported following analysis of the MR CLEAN trial registry).^24^

Large vessel occlusion and medium vessel occlusion treatment beyond the M1 and A1 segments has gradually and widely gained literature support as technology and techniques have evolved.^25-31^ Hesitancy stems from the small-caliber vessels and increasing tortuosity inherent to distal vasculature, making access increasingly challenging.^25-29^ The risk of vessel perforation with potentially devastating extravasation also increases with more distal procedures.^25-29^ However, the availability of more navigable microcatheters able to provide adequate support to deliver aspiration and mechanical thrombectomy systems has allowed for improved outcomes in select cases.^25-30^ Accordingly, continued advancements in technique and technology have facilitated reports of good functional outcome and successful recanalization in the distal vasculature of the anterior circulation.^25-27^ EVT procedures using stent-riever devices combined with aspiration or direct aspiration alone have been particularly successful in safe and effective recanalization of the insular middle cerebral artery M2 segments.^28,29^

The location of a chronic total occlusion of the right internal carotid artery is a rare finding.^32-34^ Chronic total occlusion of the internal carotid artery terminus is a commonly encountered pathology in the management of acute ischemic stroke,^20,32,33^ and acute or chronic occlusion of the cervical internal carotid artery is frequently seen in intracranial and tandem lesions.^27,32^ However, chronic total occlusion of the supraclinoid segment of the internal carotid artery distal to the anterior choroidal artery origin with reconstitution at the internal carotid artery terminus is an infrequent anatomic location.^34^

## CONCLUSION

A patient presenting with symptoms consistent with a right hemispheric acute ischemic stroke resulting from large vessel occlusion of the left A1-A2 junction underwent successful EVT with complete recanalization and resolution of clinical symptoms. This case demonstrates the importance of a high index of suspicion when evaluating atypical stroke presentations. Furthermore, the case highlights the effectiveness of EVT in the treatment of distal small-caliber vessels.

## Notes

*This article meets the Accreditation Council for Graduate Medical Education and the American Board of Medical Specialties Maintenance of Certification competencies for Patient Care and Medical Knowledge.*
